# Supplementary material for: High-Throughput Computational Screening for Bipolar Magnetic Semiconductors
Source: Research (Wash D C). 2022 Mar 15;2022:9857631. doi: 10.34133/2022/9857631 (PMC8943632; doi:10.34133/2022/9857631)
Supplement: Supplementary Materials — Table for properties of entries mp-24105, mp-1036443, mp-1190177, mp-759690, mp-560127, and mp-776070; crystal structure and density of states for Li4Ni7(OF7)2 (mp-867641), CoPtF6 (mp-556492) and Li2MnF6 (mp-754966); density of states for MnH8(NF3)2 under strain; density of states for Li4Ni7(OF7)2 (mp-867641), CoPtF6 (mp-556492), and Li2MnF6 (mp-754966); table for properties of Li2V3TeO8 of (001) oriented O1-termination slabs with different numbers of V atom layers; (001) oriented slabs of Li2V3TeO8 with different surface terminations; density of states of Li2V3TeO8 of (001) oriented O1-termination slabs; table for properties of Li-V-Te-O quaternary compounds Li2V3TeO8, LiVTeO5 and LiV3(TeO6)2. [file 9857631.f1.pdf]

Supplementary Materials: High-throughput computational screening for bipolar magnetic semiconductors

Haidi Wang[1], Qingqing Feng[2], Xingxing Li[2*] & Jinlong Yang[2*]

[1] *School of Physics, Hefei University of Technology, Hefei, Anhui 230601, China*

[2] *Hefei National Laboratory for Physical Sciences at Microscale, Department of Chemical Physics, and Synergetic Innovation Center of Quantum Information and Quantum Physics, University of Science and Technology of China, Hefei, Anhui 230026, China*

Correspondence Xingxing Li (lixx@ustc.edu.cn) & Jinlong Yang (jlyang@ustc.edu.cn)

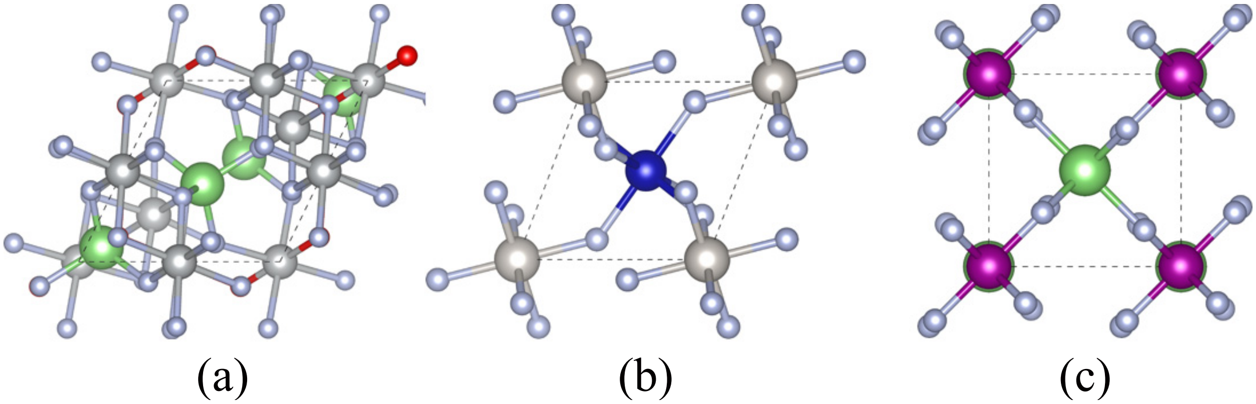

Figure S 1: (a)-(c) Crystal structure of $Li_4Ni_7(OF_7)_2$ (mp-867641), $CoPtF_6$(mp-556492) and Li_2MnF_6(mp-754966), respectively.

Table S 1: Properties of obtained compounds for BMS materials based GGA+U level simulation: Materials Project ID (MP-ID), formula, formation energy (E_f,eV/atom), energy above hull (E_{abh},eV/atom), magnetic moment per formula unit cell (m,μ_B/f.u.), minimum distance between magnetic atoms (d_{min},$\AA$), number of magnetic atom (N_m), exchange energy per magnetic atom (E_{ex},meV/atom) and space group symmetry. The spin-flip gap in valence band (Δ_1, eV), conduction band (Δ_2, eV) and the band gap (Δ_3, eV).

MP-ID	formula	E_f	E_{abh}	m	d_{min}	N_m	E_{ex}	symmetry	Δ_1	Δ_2	Δ_3
mp-24105	$Co(HO)_2$	-1.361	0.037	3.000	3.196	1	0.0084	P-3m1	0.282	0.199	2.288
mp-1036443	$CaMg_{14}CoO_{16}$	-2.939	0.036	3.000	4.320	1	0.0054	Pmmm	0.367	0.818	2.087
mp-1190177	Dy2CoTe$_2$(SO$_7$)$_2$	-2.426	0.000	3.000	5.301	1	0.0005	P-1	0.070	0.402	2.991
mp-759690	$MnH_8(NF_3)_2$	-1.611	0.089	3.000	5.876	2	0.0005	$P6_3mc$	0.069	1.335	3.114
mp-560127	K_2MnF_6	-2.915	0.001	3.000	5.834	2	0.0004	$P6_3mc$	0.068	1.322	3.153
mp-776070	$Li_4NiSn_3(PO_4)_4$	-2.365	0.087	1.999	4.845	1	0.00003	Pm	0.082	0.247	2.306

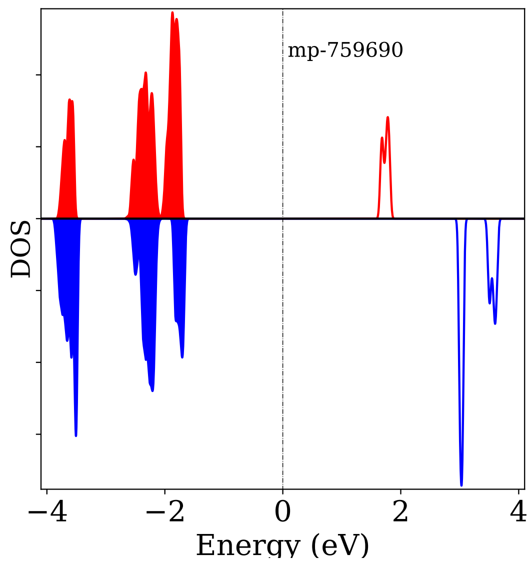

Figure S 2: Density of states for $\mathrm{MnH_8(NF_3)_2}$ (mp-759690) with 2% strain under GGA+U level theory.

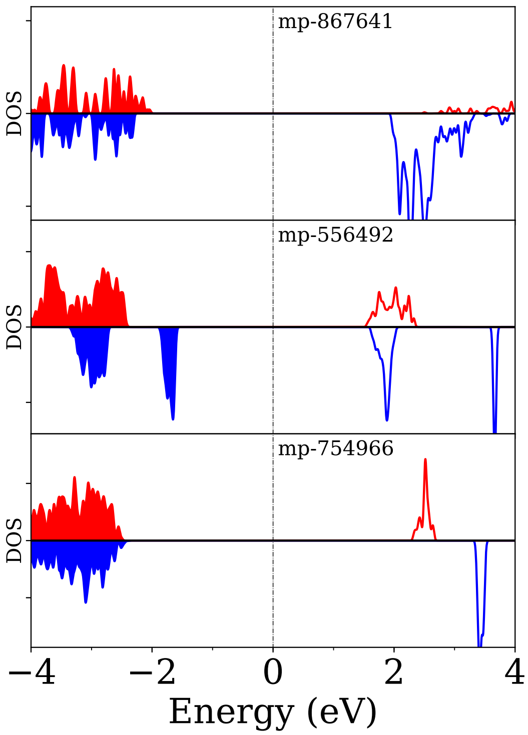

Figure S 3: Density of states for $Li_4Ni_7(OF_7)_2$ (mp-867641), $CoPtF_6$(mp-556492) and Li_2MnF_6(mp-754966), respectively.The DOSs are calculated under HSE06 level theory.

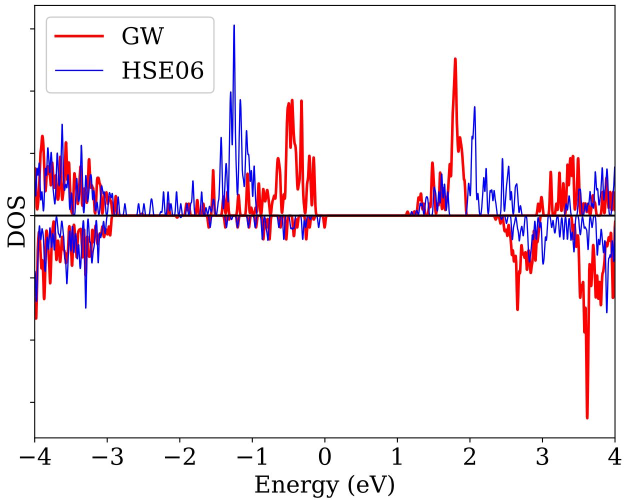

Figure S 4: A comparison of density of states for $Li_2V_3TeO_8$ (mp-771246) under HSE06 and GW level theory. The differences for spin-flip gap in valence band, conduction band and the band gap are 0.724, 0.025 and 0.005 eV, respectively.

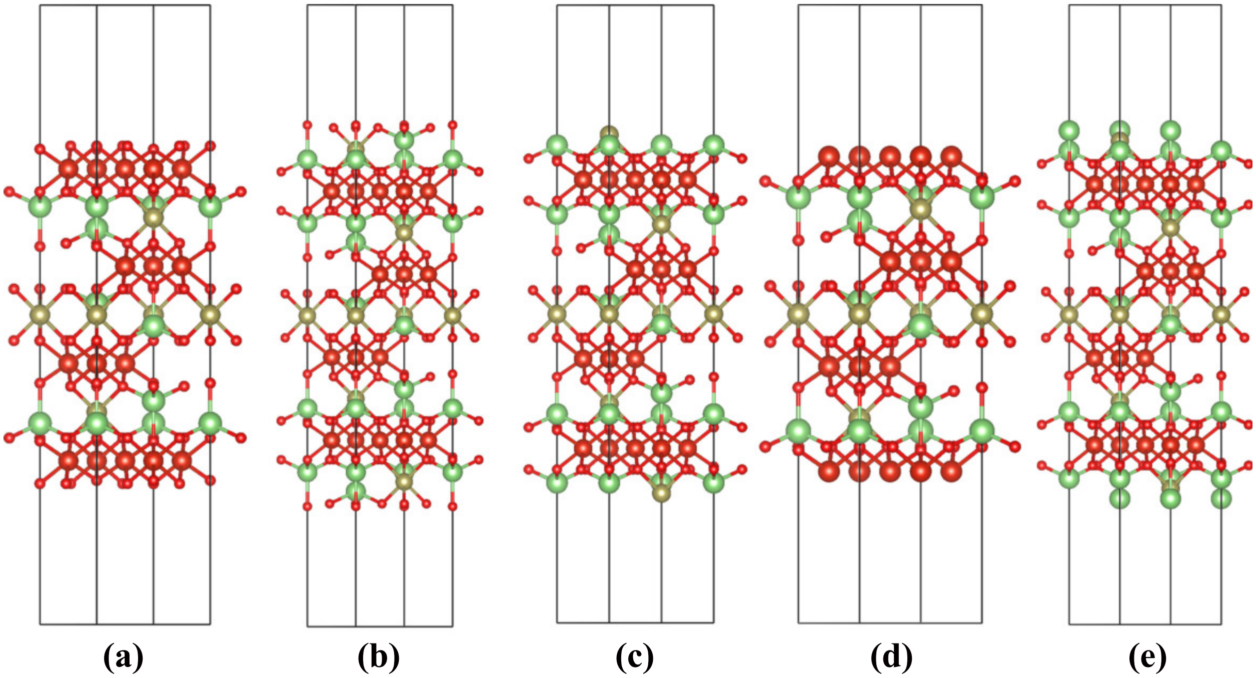

Figure S 5: (001) oriented slabs of $Li_2V_3TeO_8$ with different surface terminations. (a)-(e) are O1-, O2-, Te-, V- and Li-termination, respectively. The corresponding formation energies are -1.463 eV, -1.333 eV, -1.415 eV, -1.247 eV and -1.460 eV, respectively. The GGA+U level theory is employed.

Table S 2: Properties of $Li_2V_3TeO_8$ of (001) oriented O1-termination slabs with different number of V atom layers: layer numbers (n_{Layer}), number of total atoms (N_{atom}), formula, magnetic moment per unit cell (m,μ_B/f.u.) and classification according to the band structure of spintronics materials. HM and HSC represent half metal and half semiconductor, respectively. The GGA+U level theory is employed.

n_{Layer}	N_{atom}	formula	m	classification
2	25	$Li_2V_6TeO_{16}$	6.00	HSC
3	39	$Li_4V_9Te_2O_{24}$	9.00	HM
4	53	$Li_6V_{12}Te_3O_{32}$	16.00	BMS
5	67	$Li_8V_{15}Te_4O_{40}$	19.00	HSC
6	81	$Li_{10}V_{18}Te_5O_{48}$	28.00	HM
7	95	$Li_{12}V_{21}Te_6O_{56}$	33.00	HM
8	109	$Li_{14}V_{24}Te_7O_{64}$	38.00	HM
9	123	$Li_{16}V_{27}Te_8O_{72}$	43.00	HM
10	137	$Li_{18}V_{30}Te_9O_{80}$	48.00	HM
11	151	$Li_{20}V_{33}Te_{10}O_{88}$	53.00	BMS
12	165	$Li_{22}V_{36}Te_{11}O_{96}$	58.00	BMS

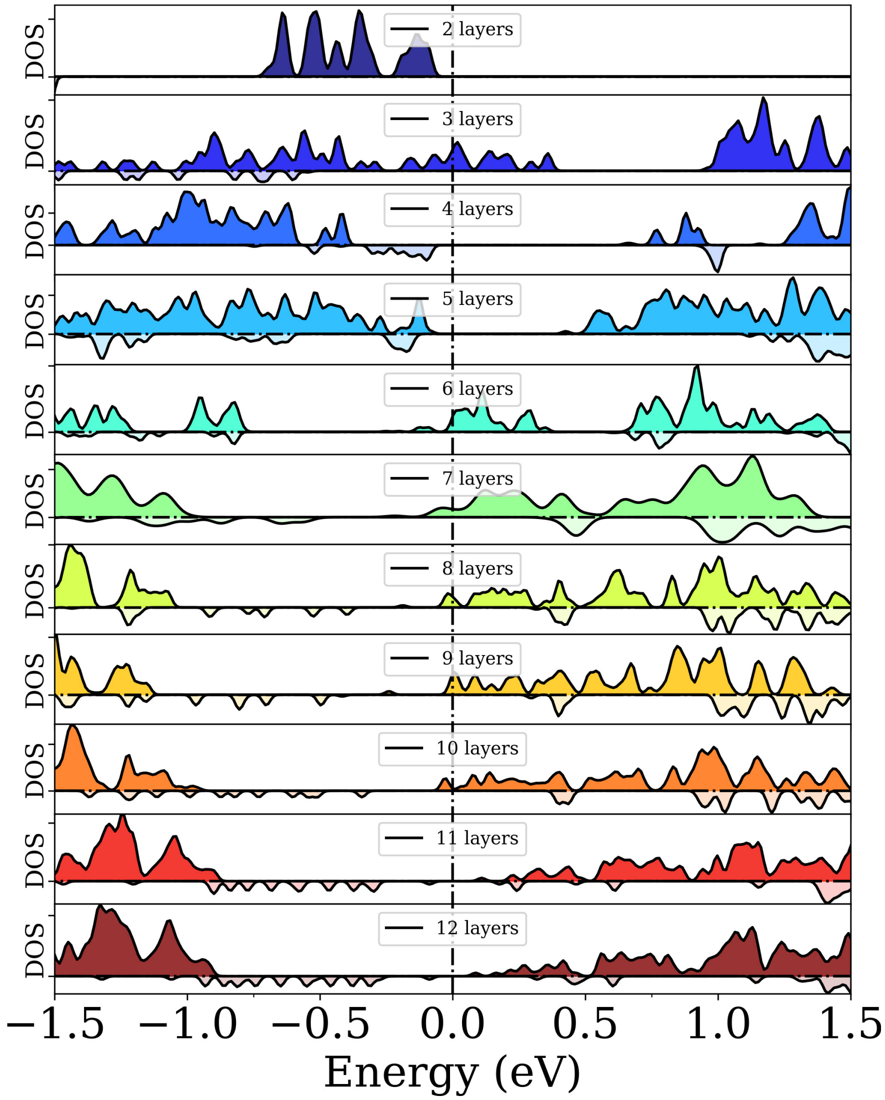

Figure S 6: Density of states of $Li_2V_3TeO_8$ of (001) oriented O1-termination slabs with number of V atom layer ranging from 2 to 12. The GGA+U level theory is employed.

Table S 3: Properties of Li-V-Te-O quaternary compounds that queried from MP database: Materials Project ID (MP-ID), formula, formation energy (E_f,eV/atom), energy above hull (E_{abh},eV/atom), reaction expression and reaction enthalpy change (kJ/mol). The reference systems are stable phases $LiTe_3$, VTe_2 and TeO_2. The GGA+U level theory is employed.

MP-ID	formula	E_f	E_{abh}	Reaction	ΔH
mp-771246	$Li_2V_3TeO_8$	-0.248	0.03	$2LiTe_3 + 3VTe_2 + 15O_2 \rightarrow 11TeO_2 + Li_2V_3TeO_8$	-6798
mp-1181006	$LiVTeO_5$	-0.118	0.06	$2LiTe_3 + 2VTe_2 + 13O_2 \rightarrow 8TeO_2 + 2LiVTeO_5$	-5777
mp-559938	$LiV_3(TeO_6)_2$	-0.132	0.10	$LiTe_3 + 3VTe_2 + 13O_2 \rightarrow 7TeO_2 + LiV_3\left(TeO_6\right)_2$	-5821
